# Effectiveness of a Large, Nation-Wide Smoking Abstinence Campaign in the Netherlands: A Longitudinal Study

**DOI:** 10.3390/ijerph16030378

**Published:** 2019-01-29

**Authors:** Sigrid A. Troelstra, Janneke Harting, Anton E. Kunst

**Affiliations:** Department of Public Health, Amsterdam UMC, University of Amsterdam, Amsterdam Public Health Research Institute, Meibergdreef 9, 1105 AZ Amsterdam, The Netherlands; j.harting@amc.uva.nl (J.H.); a.kunst@amc.uva.nl (A.E.K.)

**Keywords:** smoking cessation, intervention, temporary abstinence campaigns, Stoptober, behavioral determinants

## Abstract

From 2014, the 28-day smoking abstinence campaign ‘Stoptober’ is held in the Netherlands. Each year, more than 50,000 people participate in what has become a nation-wide collective cessation attempt. This study aims to determine the short-term effects of ‘Stoptober’ on participants’ smoking behavior and behavioral determinants. Stoptober participants completed online surveys before the start of the campaign (*n* = 6856) and three months later (*n* = 1127). Descriptive statistics and t-tests were performed to determine changes in smoking and behavioral determinants. Logistic regression analyses were used to identify differences between subgroups. After three months, 71.8% of respondents had quit smoking and consumption was reduced among sustained smokers. Cessation rates were similar for subgroups by age, sex and educational level. Cessation was positively associated with confidence and self-efficacy at baseline and negatively associated with past year quit attempts and addiction level at baseline. For quitters, we found favorable changes in attitude towards cessation related stress, social norms, social pressure to smoke, self-efficacy to quit, smoking habit strength and smoker identity. For sustained smokers, we found favorable changes in attitude towards cessation related stress, self-efficacy and smoking habit strength. These results suggest that an abstinence campaign with a wide reach in a national population may be effective in decreasing smoking prevalence and cigarette consumption among a broad range of participants.

## 1. Introduction

Tobacco use is one of the main public health threats [1]. Most smokers who are aware of the risks of tobacco use want to quit smoking [2]. However, smoking cessation can be very difficult, since dependence on tobacco comprises a cluster of behavioral, cognitive and physiological phenomena [3]. A broad range of interventions with the potential to increase success rates of cessation attempts is available, such as group behavior therapy, individual behavior counselling, telephone counselling and cessation advice in primary health care systems [4,5]. When implemented correctly, these interventions can increase quit ratios significantly, especially when combined with medication [4,5]. However, these interventions have a limited uptake because of barriers that tobacco users might experience in using cessation services, such as a lack of knowledge about the available support, perceived costs [6,7,8], ineffective recruitment [9] and inflexible support [10].

Given such barriers in accessibility and acceptability, it is important to develop interventions that are able to reach a large number of smokers willing to quit. The Stoptober campaign can do so at relatively little cost. This national 28-day smoking cessation intervention aims to decrease smoking prevalence and tobacco use at national levels by challenging smokers to stop smoking collectively for 28 days during the month October and by asking non-smokers to encourage and support participants [11]. By having a predetermined quit date and an attainable goal to stop smoking for a limited time period, a substantial part of smokers might be willing to participate and be successful [11]. The Stoptober campaign is based on social contagion theory, SMART (i.e., Specific, Measurable, Attainable, Realistic and Time-sensitive) goal setting and PRIME (i.e., Plans, Responses, Impulses, Motives and Evaluations) theory [11,12,13,14]. Before the start of the campaign, Stoptober uses traditional and new mass media channels to create a mass quitting trigger and actively support a social movement around stopping smoking. Stoptober challenges smokers to set an intermediary goal, i.e., to stop smoking for a time-limited period. This goal may be relatively easy to achieve, and once achieved, it could substantially increase the chances of becoming a permanent non-smoker. During the campaign, Stoptober offers an elaborate support package, consisting of motivational Twitter messages, role models that act as Stoptober ambassadors, video diaries of fellow participants, an active Facebook community, social media profile logos for participants to notify friends and family of their participation and a Stoptober app. An overview of the key psychological principles and program components of the Stoptober 2016 campaign in the Netherlands is presented in Table S1.

The Stoptober temporary abstinence campaign had more than 215,000 participants in the United Kingdom in 2015 [15]. In the Netherlands in 2016, Stoptober had more than 50,000 registered participants. A study on the effectiveness of Stoptober in 2012 in England found a 4.15% increase in quit rates on a national level during October 2012 [11]. Furthermore, it concluded that Stoptober is a cost-effective intervention that yields a substantial return in behavior change and public health impact. However, no information was available on maintenance of these quit attempts and the effectiveness for different groups of participants. A preliminary study on the effects of the Dutch Stoptober campaign of 2015 found a quit rate of 67% among its respondents three months after the start of the campaign [16]. According to this study, participants who were male, younger or lower educated were more likely to succeed in not smoking for 28 days during Stoptober [16]. However, this research was conducted retrospectively, which severely limits the validity of the results.

Therefore, the aim of this study is to determine the short-term effects of the Stoptober 2016 campaign on smoking behavior and behavioral determinants. Furthermore, we are interested in potential changes in behavioral determinants of smoking cessation. The specific objectives were to study (1) the effect of the intervention on smoking behavior after three months; (2) the effectiveness for different subgroups of smokers; and (3) the effect of the intervention on behavioral determinants. To our knowledge, this is the first evaluation of Stoptober that adopted a prospective cohort design and included behavioral determinants of smoking cessation.

## 2. Materials and Methods 

### 2.1. Design

This observational study had a prospective cohort design. Respondents were followed through subsequent surveys, which were distributed online to participants: A baseline survey before the intervention in September 2016 and a three-month follow-up survey in December 2016 and January 2017.

The baseline survey consisted of questions about demographic characteristics (age, sex, educational level) and smoking behavior (frequency, addiction level, former cessation attempts, former Stoptober participation). Additionally, in the baseline survey we asked respondents about their determination to quit and the confidence they had in succeeding in their quit attempt. In the three-month follow-up survey, we asked respondents whether they remained abstinent during the 28 days of Stoptober and what their current smoking behavior was (smoking status, frequency). In both the baseline and the three-month follow-up survey, we asked questions about behavioral determinants (attitude towards non-smoking, non-smoking social norm, self-efficacy, smoking habit strength, non-smoking identity). 

### 2.2. Participants

In total, around 53,000 individuals subscribed online as a Stoptober participant. All participants who had subscribed before September 28th (*n* = 19,363) were contacted via email with an invitation to participate in the surveys. Respondents who reported in the baseline survey that they were willing to participate were invited for a subsequent survey (*n* = 6856). For both survey invitations, one reminder was sent to respondents who did not complete the survey yet. The three-month follow-up survey was answered by 1159 participants. Participants were excluded if we were unable to link a participant to the baseline survey (*n* = 21) and if they did not disclose their current smoking status (*n* = 11). 1127 participants were included in this study.

### 2.3. Demographic Variables

Age was categorized into <45, 45–59, >59 years, and educational level was categorized into low, medium, and high educational level. Low educational level included primary education, lower vocational and lower secondary education; medium educational level included intermediate vocational and higher secondary education; high educational level included higher vocational education and university [17].

### 2.4. Smoking Variables

Our main outcome was smoking cessation three months after the start of the Stoptober campaign, which was measured by asking respondents: “Do you currently smoke?”, with answer options: “yes” and “no”. Additionally, abstinence during the campaign was measured in retrospect by asking respondents: “Did you manage to complete Stoptober by not smoking for 28 days?”, with answer options: “yes” and “no”. Average daily cigarette consumption was measured by asking: “How many cigarettes do you smoke on average each day?” Answers were categorized into “0–1”, “2–5”, “6–10”, “11–15”, “16–20” and “more than 20” cigarettes per day. Being a heavy smoker was operationalized as smoking twenty or more cigarettes per day on average. To determine cigarette addiction smokers were asked: “How soon after you wake up do you usually smoke your first cigarette of the day?”. Categories were divided into “within 5 min”, “between 5 to 30 min”, “between 30 to 60 min” and “after 60 min”. Having a strong smoking addiction was operationalized as smoking the first cigarette of the day within 5 minutes after waking up, being the strongest predictor for short- and long-term quit success [18].

### 2.5. Behavioral Determinants

Individual items for each concept are available in the Supplementary Files (Table S2). Concepts from the Theory of Planned Behavior (attitude, social norm and self-efficacy) were included in our survey [19]. Positive attitude towards non-smoking was measured by five items (α = 0.804) [20]. Social norm towards non-smoking was measured by three items (α = 0.728) [16]. Self-efficacy towards non-smoking was measured by seven items (α = 0.803) [16,20]. Two single items did not fit within a scale: Social pressure to smoke and negative attitude towards cessation related stress.

Four other behavioral determinants that might be associated with smoking cessation were measured in the surveys. Smoking habit strength was measured according to the automaticity subscale and included three items (α = 0.819) [21]. Non-smoking identity was measured by two items (α = 0.717) [22,23]. Confidence in succeeding to stop smoking [24] and determination to stop smoking [25] were measured in the baseline survey with a single item. 

If scale items for positive attitude towards non-smoking or self-efficacy towards non-smoking were missing or not applicable for a participant, scales were still composed for that individual on the condition that at least 75% of the items on that scale were completed. In order to be able to distinguish clearly between participants who scored lower or higher on certain variables, Likert scale variables were dichotomized based on the cut-off value nearest to the average score of that variable.

### 2.6. Data Analysis

We described proportions of successful participants for demographic characteristics, smoking behavior and scores on behavioral determinants as descriptive analyses. To determine the effect of the Stoptober campaign on smoking behavior, we tested for differences in smoking prevalence and the proportion of heavy smokers. Next, to determine which participants were most likely to have quit smoking after three months, we performed univariable and multivariable logistic regression analyses. In the multivariable regression analyses, all variables on demographics, smoking and behavioral determinants were entered into the regression model.

Subsequently, to determine the effect of the Stoptober intervention we analyzed changes in scores on behavioral determinants with paired t-tests. A sensitivity analysis was performed in which the determinants of smoking behavior were added to the model as continuous variables. We observed no differences in the association between determinants and having stopped smoking three months after the start of the campaign. Finally, we performed a non-response analysis to determine the selectiveness of drop-outs. All analyses were performed using SPSS (version 24) (IBM, Armonk, NY, USA).

The AMC Medical Ethics Review Committee reviewed the study proposal and concluded that the Medical Research Involving Human Subject act (WMO) does not apply to this study and that an official approval by this committee was not required (letter W16_327). 

The datasets used and/or analyzed during the current study are available from the corresponding author on reasonable request.

## 3. Results

The participants of the three-month follow-up survey were mainly women (71.8%) (Table 1) and on average they were 45 years old. 25.3% of the respondents had a low educational level, 42.5% a medium level and 32.1% a high educational level. Of the respondents, 19.3% had participated in Stoptober before and 41.4% attempted to quit smoking in the past year. At baseline, 35.2% were heavy smokers and 20.4% were heavily addicted smokers. Before the intervention, participants smoked on average 15.9 cigarettes a day. 

After three months, 71.8% of the respondents stopped smoking. Additionally, 69.3% of the respondents reported in retrospect that they did not smoke during the 28 days of Stoptober. Among the participants who reported to smoke at the time of the three-month follow-up survey, the proportion of heavy smokers (more than 20 cigarettes a day) significantly declined from 25.5% to 5.3% (Table 2).

Cessation rates were similar for subgroups defined in terms of age, sex and educational level. The group-specific rates ranged from 66.9% to 74.3% (Table 1, upper part). The differences between these groups were not statistically significant. Respondents who were heavy smokers were less likely to have quit smoking after three months (Table 1). However, these differences were not significant in multivariable analyses. Respondents were significantly less likely to have stopped smoking when they participated in the Stoptober program before (OR (odds ratio) 0.53, 95% CI (confidence interval) 0.38–0.73), when they attempted to stop smoking in the past year (OR 0.68, 95% CI 0.51–0.89) and when they were heavily addicted smokers (OR 0.67, 95% CI 0.47–0.96).

Respondents were more likely to have stopped smoking after three months when at baseline they scored higher on determination to quit, confidence in succeeding to quit, positive attitude towards non-smoking or self-efficacy and lower on negative attitude towards non-smoking (stress), social pressure to smoke or smoking habit strength (Table 3). In multivariable analyses, significant associations between confidence in succeeding to quit (OR 2.03, 95% CI 1.41–2.93) and self-efficacy (OR 1.34, 95% CI 1.00–1.79), and having stopped smoking after three months were found. 

For respondents who quit smoking, we observed significant decreases in positive attitude towards non-smoking and social pressure to smoke and significant increases in social norm towards non-smoking, self-efficacy and non-smoking identity after three months (Table 4). For respondents who remained smokers, we found significant decreases for positive attitude towards non-smoking, negative attitude (stress) and smoking habit strength and a significant increase for self-efficacy.

Since the drop-out rate at the three-month follow-up survey was 83.6%, we conducted a non-response analysis (Table S2). We found that compared to respondents who only completed the baseline survey, respondents who completed the three-month follow-up survey differed significantly in terms of age, education level, former participation in Stoptober, being a heavy smoker, being an addicted smoker, determination, confidence, social norm, self-efficacy, habit and identity. Of these variables, former participation of the campaign was negatively associated with having quit smoking at three months and having a higher addiction level, more confidence and a higher self-efficacy to quit were positively associated with having quit smoking at three months (Table 1 and Table 3). The other variables were not associated with having quit smoking at three months.

## 4. Discussion

### 4.1. Summary of Findings

We found that three months after the start of a nation-wide smoking cessation campaign, 71.8% of the participants that responded to the follow-up survey had quit smoking, while tobacco consumption rates decreased among participants who still smoked after the campaign. Smoking cessation rates were similar for subgroups defined in terms of age, sex and educational level. Smoking cessation after three months was positively associated with confidence and self-efficacy and negatively associated with past year quit attempts and addiction level. For respondents who quit smoking, attitude towards cessation related stress, social norms, social pressure to smoke, self-efficacy and non-smoking identity changed favorably. For respondents who still smoked attitude towards cessation related stress, smoking habit strength and self-efficacy changed favorably. 

### 4.2. Evaluation of Study Limitations

The particularities of this nation-wide cessation campaign, in which participants decide to participate at the very last moment and show varying levels of involvement, poses particular difficulties to its evaluation. The main limitation of this study is its low response rate at the follow-up. The response rate in the three-month follow-up survey was 16.4%. It can be expected that attrition was not random, but that participants who stopped smoking were more likely to participate in the follow-up surveys compared to participants who still smoked. If attrition is indeed selective, the results may be subject to selection bias. The non-response analysis found substantial differences between baseline respondents who did and did not participate in the three-month follow up survey for demographic, smoking and behavioral variables. 

We could also relate risk of attrition to the respondents’ abstinence during the 28 days of the Stoptober campaign, as measured in an additional survey immediately after the campaign. We found that participants who succeeded in abstaining during the campaign were three times as likely to respond in the three-month follow-up (not reported in this paper). Assuming that this three-fold difference applies to all Stoptober participants at the time of the three-month follow-up, we estimated that that the three-month follow-up survey, with an overall response rate of 16.4%, was answered by 9.5% of the sustained smokers versus 24.0% of those who had quit smoking. Further, we quantified the extent to which such a differential response had resulted in an overestimation of quit rates. Adjusting for this overestimation, we estimated that that the observed quit rate of 71.8% had to be adjusted downwards to 50.2%. Therefore, our best estimate of the three-month quit rate is around 50%. However, further research, with higher response rates, also from respondents who relapsed, is needed to assess the long-term effectiveness of this campaign. 

A potential limitation of this study is that data are based on self-reporting. Evidence on the sensitivity and specificity of self-reported smoking status is mixed. A meta-analysis comparing self-reported smoking status with biochemical validation found a generally high sensitivity and specificity on self-reported smoking status [26]. In contrast, a more recent systematic review found underestimation of smoking prevalence and varying sensitivity levels [27]. To the extent that sensitivity was reduced in our sample, we would have overestimated quit rates three months after the program.

A further limitation of this study relates to the instruments that we used to measure behavioral determinants. We aimed to use as much as possible validated questionnaires, but those available in the scientific literature did not always suit the topic of our study (smoking cessation). Therefore, we used new questions, based on Likert scales, to measure determination, confidence, social norm and social pressure, self-efficacy and identity. Furthermore, due to the need to keep the survey as short as possible, we used only a few items of the validated scales on habit strength and smoker identity. Cronbach’s alphas were above 0.7 and indicated a high internal consistency. Despite this, changes in or relationships with these determinants may be underestimated due to inaccurate measurement.

### 4.3. Interpretation of Results

We estimated that Stoptober participants had a quit rate of about 50% after three months. This is high compared to the estimated 10 to 20% three-month abstinence ratio reported for unsupported quitters [28]. This low abstinence ratio for unsupported quitters is largely due to quitters who relapse during the first month of their cessation attempt. Stoptober may make a difference in this first critical month, since 69.3% of the respondents succeeded in remaining abstinent for one month during Stoptober. The effect of smoking cessation interventions is commonly measured after one year. According to one study, half of the participants who stopped smoking after three months will remain abstinent after one year [29]. Applied to our case, it would mean that our estimated quit rate of 50% after three months would correspond to a quit rate of about 25% after one year. According to a systematic review, the success percentage after one year for smokers who quit without support is below 5% [28]. In general, it is suggested that interventions causing a decrease in smoking prevalence of 5 to 10% after one year might be effective in comparison to no intervention [28]. Against this yardstick, Stoptober can probably be considered an effective intervention, also at the population level. A 25% quit rate would imply that of the 50,000 participants to the 2016 Stoptober campaign, more than 10,000 smokers would have remained smoke-free after one year.

In comparison with tobacco users in the general Dutch population, the respondents in our study were more often of older age [30] and had a higher tobacco consumption rate [31], while the education level was similar [30]. In general, older individuals and heavier tobacco users are less likely to quit smoking [32,33]. Thus, Stoptober achieved a wide reach not only among the general national population, but including among population groups of particular concern. This high generalized reach could be achieved at relatively low costs compared to smoking cessation interventions focused on individual quitters. In its design, Stoptober may have circumvented well-known barriers to participation in smoking cessation services, such as a lack of knowledge about the intervention [6], perceived costs to participants [34] and insufficient attention to social support [35].

The Stoptober campaign was less effective for participants who had attempted to stop smoking in the past year and for those who participated in Stoptober before. These observations are in accordance with the findings of studies on smoking cessation maintenance that smokers who have recently attempted to quit are more likely to try again but also more likely to relapse [36]. A potential consequence could be that the population of smokers who can quit easily slowly depletes. In this case, the effectiveness of Stoptober might decrease in the upcoming years, since the remaining and recurrent participants are expected to be less successful each year. 

The high three-month quit rates of Stoptober participants could at least partly be explained by its influence on behavioral determinants, specifically on self-efficacy. We observed a small increase in self-efficacy for those who remained smoking for three months and a much larger increase in self-efficacy for non-smokers. Moreover, we found that participants were more likely to remain smoke-free after three months when they had more confidence in their quit attempt and a higher self-efficacy before the start of the campaign. This is in line with previous studies, where high self-efficacy was found to be a strong predictor of quit success [37,38,39]. Evidence from a systematic review on the relation between confidence in succeeding and quit success is inconsistent [39]. Participation in Stoptober was related to positive change in only part of behavioral determinants. Opposite trends were found for attitude towards non-smoking. This could possibly be explained by a more realistic perception of the difficulties of remaining a non-smoker after an initial successful quit attempt.

The organization of the Stoptober campaign could have influenced self-efficacy in several ways. Essential to the campaign is a focus on a SMART goal for quitting smoking for 28 days, which is found to be acceptable and reachable to most of the participating smokers [11]. Furthermore, self-efficacy of participants can be enhanced by the collective aspect of a cessation attempt through Stoptober and the possibility to share experiences and learn from each other supplies participants with peer support [11]. In addition, the Stoptober campaign provides participants with positive stories of former quitters, and it uses its Facebook page and app to disseminate tips to remain abstinent. 

## 5. Conclusions

Given their large reach and relatively low costs, temporary abstinence campaigns such as Stoptober have a significant potential to contribute to tackling smoking at national and local levels. Our results suggest that these population-based interventions are effective in increasing quit success among those willing to quit, and reducing tobacco consumption among those yet unable to quit. Further evaluations with high response rates are needed to assess the long-term effectiveness of this campaign and to explore ways to enhance the reach and effectiveness at different national and local settings.

## Figures and Tables

**Table 1:** Demographic characteristics and smoking variables and their association with not smoking after three months (*n* = 1127).

Variables	% Participants (N) ^1^	% Stopped Smoking after 3 Months (N)	Odds Ratio (95% CI) ^2^	
			Univariable Regression	Multivariable Regression ^3^
**Demographics**				
Gender				
Men	28.2 (318)	71.7 (228)	1.00	1.00
Women	71.4 (805)	71.8 (578)	1.01 (0.75–1.34)	1.00 (0.76–1.37)
Missing	0.4 (4)			
Age				
<40	34.4 (388)	73.2 (284)	1.00	1.00
40–54	39.6 (446)	73.8 (329)	1.03 (0.76–1.40)	1.11 (0.81–1.52)
>55	26.0 (293)	66.9 (196)	0.74 (0.53–1.03)	0.86 (0.61–1.23)
Missing	-			
Education				
Low ^4^	25.3 (285)	69.5 (198)	1.00	1.00
Medium ^5^	42.5 (479)	71.2 (341)	1.09 (0.79–1.50)	0.92 (0.65–1.29)
High ^6^	32.1 (362)	74.3 (269)	1.27 (0.90–1.80)	1.11 (0.77–1.60)
Missing	0.1 (1)			
**Smoking variables**				
Former participation campaign				
No	80.6 (908)	75.0 (681)	1.00	1.00
Yes	19.3 (217)	58.1 (126)	0.46 (0.34–0.63) *	0.53 (0.38–0.73) *
Missing	0.2 (2)			
Cessation attempt in past year				
No	58.4 (658)	76.4 (503)	1.00	1.00
Yes	41.4 (467)	65.1 (304)	0.58 (0.44–0.75) *	0.68 (0.51–0.89) *
Missing	0.2 (2)			
Heavy smoker				
No	64.6 (728)	74.7 (554)	1.00	1.000
Yes	35.2 (397)	66.5 (264)	0.67 (0.51–0.88) *	0.77 (0.57–1.04)
Missing	0.2 (2)			
Addicted smoker				
No	79.6 (897)	74.0 (664)	1.00	1.00
Yes	20.4 (230)	63.0 (145)	0.60 (0.44–0.81) *	0.67 (0.47–0.96) *
Missing	-			

* significant difference at α ≥ 0.05, ^1^ N: number, ^2^ CI: confidence interval, ^3^ Corrected for all demographic and smoking variables presented in this table. ^4^ Primary education (basisschool), lower secondary education (BBL, KBL, VMBO), lower vocational education (LBO), ^5^ Middle or higher secondary education (HBS, HAVO, VWO), middle vocational education (MTS, MULO, MBO), ^6^ Higher vocational education (HBO), university.

**Table 2:** Changes in smoking prevalence and daily cigarette consumption after three months (N = 1127).

Smoking Variables	Baseline % (N) ^1^	After 3 Months % (N)
**Smoking cessation after 3 months (N = 1127)**	100.0 (1127)	71.8 (809)
**Daily cigarette consumption sustained smokers (N = 318)**		
0–1	0.6 (2)	6.9 * (22)
2–5	4.7 (15)	19.2 * (61)
6–10	23.6 (75)	46.2 * (147)
11–15	23.0 (73)	14.8 * (47)
16–20	22.3 (71)	7.5 * (24)
>20	25.5 (81)	5.3 * (17)
Missing	0.3 (1)	-

* significant difference at α ≥ 0.05 compared to baseline survey. ^1^ N: number.

**Table 3:** Behavioral determinants at baseline and their association with not smoking after three months (N = 1127).

Behavioral Determinants ^1,2^	% of Participants (N)	% Stopped Smoking after 3 Months (N)	Odds ratio (95% CI) ^3^	
			Univariable Regression	Multivariable
				Regression ^4^
**Determination to quit**				
Lower score (≤4)	11.9 (134)	64.9 (87)	1	1
Higher score (>4)	88.0 (992)	72.7 (721)	1.44 (0.98–2.10)	0.84 (0.53–1.35)
Missing	0.1 (1)			
**Confidence in succeeding to quit smoking**				
Lower score (<4)	22.7 (256)	61.3 (157)	1	1
Higher score (≥4)	77.1(869)	74.8 (650)	1.87 (1.39–2.51) *	2.03 (1.41–2.93) *
Missing	0.2 (2)			
**Positive attitude towards non-smoking**				
Lower score (<4.5)	20.6 (232)	71.6 (166)	1	1
Higher score (≥4.5)	79.4 (895)	71.8 (643)	1.01 (0.74–1.40)	0.92 (0.65–1.30)
Missing	-			
**Negative attitude towards non-smoking (stress)**				
Lower score (<4)	35.0 (395)	73.9 (292)	1	1
Higher score (≥4)	65.0 (732)	70.6 (517)	0.85 (0.64–1.12)	0.96 (0.71–1.29)
Missing	-			
**Social norm towards non-smoking**				
Lower score (<3.3)	25.5 (287)	69.3 (199)	1	1
Higher score (≥3.3)	74.5 (840)	72.6 (610)	1.17 (0.88–1.57)	1.22 (0.89–1.68)
Missing	-			
**Social pressure to smoke**				
Lower score (<3)	74.8 (843)	73.5 (620)	1	1
Higher score (≥3)	25.2 (284)	66.5 (189)	0.72 (0.54–0.96) *	0.80 (0.59–1.10)
Missing	-			
**Self-efficacy towards non-smoking**				
Lower score (<2.5)	46.5 (524)	67.0 (351)	1	1
Higher score (≥2.5)	53.1 (598)	75.9 (454)	1.55 (1.20–2.02) *	1.34 (1.00–1.79) *
Missing	0.4 (5)			
**Smoking habit strength ^5^**				
Lower score (<3.7)	30.9 (348)	73.9 (257)	1	1
Higher score (≥3.7)	68.9 (776)	70.9 (550)	0.86 (0.65–1.15)	0.96 (0.70–1.32)
Missing	0.3 (3)			
**Non-smoking identity**				
Lower score (<3)	25.8 (291)	68.7 (200)	1	1
Higher score (≥3)	74.2 (836)	72.8 (609)	1.22 (0.91–1.63)	0.85 (0.63–1.15)
Missing	-			

* significant difference at α ≥ 0.05 compared to baseline survey, ^1^ Individual items for each scale are presented in Table S2, ^2^ If scale items for positive attitude towards non-smoking or self-efficacy towards non-smoking were missing or not applicable for a participant, scales were still composed for that individual on the condition that at least 75% of the items on that scale were completed, ^3^ CI: confidence interval, ^4^ Corrected for all demographic characteristics and smoking variables presented in Table 1 and all behavioral determinants presented in this table, ^5^ Likert scale items for smoking habit strength were only reported by those who were current smokers at the three-month follow-up survey.

**Table 4:** Changes in score on Likert scales (1–5) of behavioral determinants for participants who stopped and did not stop smoking after three months.

	Stopped Smoking after 3 Months (N = 809)			Smoked after 3 Months (N = 318)		
	Baseline	Follow-Up	Δ (N)	Baseline	Follow-Up	Δ (N)
	M ± SD ^1^	M ± SD		M ± SD	M ± SD	
**Change after 3 months (M ± SD)**						
Positive attitude towards non-smoking	4.52 ± 0.62	4.40 ± 0.57	−0.12 * (783)	4.48 ± 0.57	3.41± 0.61	−1.07 * (301)
Negative attitude towards non-smoking (stress)	3.57 ± 1.00	3.68 ± 1.08	0.11 * (789)	3.67 ± 1.02	3.13 ± 1.18	−0.54 * (302)
Social norm towards non-smoking	3.42 ± 0.86	3.71 ± 0.77	0.29 * (779)	3.39 ± 0.83	3.44 ± 0.78	0.05 (296)
Social pressure to smoke	2.00 ± 0.95	1.79 ± 0.84	−0.21 *16 (779)	2.13 ± 0.96	2.20 ± 0.95	0.07 (296)
Self-efficacy towards non-smoking	2.58 ± 0.72	4.05 ± 0.72	1.47 * (783)	2.40 ± 0.71	2.81 ± 0.85	0.41 * (302)
Smoking habit strength	3.58 ± 0.90	-	-	3.63 ± 0.91	3.32 ± 0.98	−0.31 * (286)
Non-smoking identity	3.06 ± 0.80	3.24 ± 0.82	0.18 * (787)	3.07 ± 0.79	3.10 ± 0.80	0.03 (301)

* significant difference at α ≥ 0.05 compared to baseline survey, ^1^ M: mean, SD: standard deviation.

## Supplementary Materials

The following are available online at http://www.mdpi.com/1660-4601/16/3/378/s1, Table S1. Key psychological principles underpinning Stoptober’s key psychological principles and corresponding program components of the Stoptober 2016 campaign in the Netherlands, Table S2: Comparison of baseline variables between respondents who only answered the baseline survey (N = 5728) and respondents of the three-month follow-up (N = 1127) for demographics, smoking variables and behavioral determinant scores on a scale of 1 (strongly disagree) to 5 (strongly agree).
