# Supplementary material for: Effectiveness of a Large, Nation-Wide Smoking Abstinence Campaign in the Netherlands: A Longitudinal Study
Source: Int J Environ Res Public Health. 2019 Jan 29;16(3):378. doi: 10.3390/ijerph16030378 (PMC6388192; doi:10.3390/ijerph16030378)
Supplement: Supplementary file 1 [file ijerph-16-00378-s001.pdf]

Table S1. Key psychological principles underpinning Stoptober's key psychological principles and corresponding program components of the Stoptober 2016 campaign in the Netherlands.

Psychological Principle [1]	Program Components
**Social contagion**<br>Social networks can facilitate the spread of attitudes and behavior and increase the reach and intensity of a message. Stoptober used traditional and new mass media channels to create a mass quitting trigger and actively support a social movement around stopping smoking. Positive messaging was used to build engagement, enhance message dissemination and normalize quitting behavior. Stoptober aimed to increase support for the campaign, and to motivate smokers to participate and encourage others to do so too, since interconnected groups of smokers often try to quit together.	Press exposure, television and radio broadcasts include the message that Stoptober is coming up and all smokers should participate and try to quit smoking temporarily on the same date. Non-smokers are encouraged to support smokers in their participation.<br><br>The Stoptober camper travels to various parts of the country to reach a large number of people. The camper personnel provide information about smoking cessation and encourage participants to take part in Stoptober.
**SMART goals**<br>SMART (Specific, Measurable, Attainable, Realistic and Time-sensitive) goals help people to achieve a difficult behavior changes, such as stopping smoking for good. Therefore, Stoptober challenged smokers to set an intermediary goal, i.e., to stop smoking for a time-limited period. This goal may be relatively easy to achieve, and once achieved, it substantially increases the chances of becoming a permanent non-smoker.	Set time and duration of collective cessation attempt. Stoptober challenges smokers not to smoke for 28 days during the month October.
**PRIME theory**<br>PRIME theory is a comprehensive theory of motivation. Behavior is determined from moment to moment by a wide variety of motivational inputs, while the motivational system is unstable and requires constant balancing of inputs to maintain a certain behavior (e.g., smoking). Programs that aim to achieve behavior change, e.g., quitting smoking, should offer a range of support that triggers the whole motivational system rather than single elements.[2] This support should both weaken the motivational powers that cause the behavior (e.g., smoking) and create new sources of desire and control to refrain from that behavior (e.g., quitting smoking). Therefore, Stoptober offered an elaborate support package, consisting of Twitter messages, ambassadors, video diaries, a Facebook community, social media profile logos and an app. This support package aims to decrease the motivation to smoke and create new desires to quit smoking.	Subscription on the website to have access to the Stoptober app, a free magazine and newsletters<br>Email messages with tips, progress, motivational messages and news<br>Bracelets to wear during the campaign to remind participants not to smoke and demonstrate their commitment to others<br>Twitter account wherein Stoptober frequently posts positive and encouraging messages for the participants<br>Well-known ambassadors participate in Stoptober, try to gain publicity for the program through their media presence and serve as examples for other participants.<br>Video diaries of Stoptober participants on YouTube and Facebook.<br>Facebook page wherein Stoptober frequently posts positive and encouraging messages for the participants and where they can share their accomplishments and struggles. Participants can share tips, compliments and encouragement.<br>Stoptober logos for Facebook profile pictures to notify friends and family of their participation.<br>Stoptober app keeps track of abstinence, amount of money saved and number of unsmoked cigarettes. Participants can earn achievement badges and press an 'emergency' button to help with cravings.

1 Brown J, Kotz D, Michie S, Stapleton J, Walmsley M, West R. How effective and cost-effective was the national mass media smoking cessation campaign 'Stoptober'? Drug Alcohol Depend. 2014;135(100):52-8.

2 West R, Brown J. Theory of Addiction: Wiley; 2013.

Table S2. Comparison of baseline variables between respondents who only answered the baseline survey (N = 5728) and respondents of the three-month follow-up (N = 1127) for demographics, smoking variables and behavioral determinant scores on a scale of 1 (strongly disagree) to 5 (strongly agree).

	**Variables**	**Baseline survey only respondents**	**3 month follow-up respondents**
**Demographics % (N)**[1]	**Gender**		
	Men	27.7 (1589)	28.2 (318)
	Women	71.9 (4116)	71.4 (805)
	Missing	0.4 (23)	0.4 (4)
	**Age**		
	<40	42.2 (2415)	34.4* (388)
	40-54	39.2 (2247)	39.6* (446)
	>55	18.6 (1066)	26.0* (293)
	Missing	-	-
	**Education**		
	Low [3]	26.7 (1528)	25.3* (285)
	Medium [4]	46.4 (2657)	42.5* (479)
	High [5]	26.7 (1529)	32.1* (362)
	Missing	0.2 (14)	0.1 (1)
**Smoking variables % (N)**	**Former participation campaign**		
	No	84.5 (4842)	80.6* (908)
	Yes	15.3 (874)	19.3* (217)
	Missing	0.2 (12)	0.2 (2)
	**Cessation attempt in past year**		
	No	56.0 (3206)	58.4 (658)
	Yes	44.0 (2519)	41.4 (467)
	Missing	0.1 (3)	0.2 (2)
	**Heavy smoker**		
	No	56.9 (3,257)	64.6* (728)
	Yes	43.1 (2,471)	35.2* (397)
	Missing	-	0.2 (2)
	**Addicted smoker**		
	No	74.4 (4261)	79.6* (897)
	Yes	25.6 (1467)	20.4* (230)
	Missing	-	-
**Behavioral determinants M ± SD**[2] **(N)**	**Determination**		
	I am determined to stay abstinent for 28 days	4.20 ± 1.03 (5725)	4.37 ± 0.95 * (1126)
	**Confidence**		
	I am confident I will stay abstinent for 28 days	3.87± 0.96 (5725)	4.11 ± 0.92* (1125)
	**Positive attitude**		
	Average score of scale [α 0.804]	4.53 ± 0.54 (5728)	4.51 ± 0.60 (1127)
	Not smoking gives you a better breath	4.50 ± 0.74)	4.50 ± 0.77
	Not smoking makes you fitter	4.48 ± 0.73	4.44 ± 0.79
	Not smoking is pleasant for my environment	4.37 ± 0.82	4.34 ± 0.86
	Not smoking saves money	4.74 ± 0.62	4.70 ± 0.68
	Not smoking is a good example for children	4.58 ± 0.73	4.56 ± 0.76
	**Negative attitude (stress)**		
	Not smoking causes stress	3.61 ± 1.00 (5728)	3.61 ± 1.01 (1127)
	**Social norm**		
	Average score of scale [α 0.819]	3.32 ± 0.88 (5728)	3.42 ± 0.84 * (1127)
	Most people I know don't smoke	3.19 ± 1.14	3.33 ± 1.11
	Most people I know think it's normal not to smoke	3.35 ± 1.03	3.50 ± 0.99
	People I know want me to stop smoking	3.41 ± 1.10	3.43 ± 1.08

**Social pressure**		
People I know pressure me to keep smoking	2.06 ± 0.94 (5728)	2.04 ± 0.96 (1127)
**Self-efficacy**		
Average score of scale [α 0.803]	2.45 ± 0.73 (5705)	2.52 ± 0.72 * (1122)
It is easy not to smoke at certain days	2.30 ± 1.19	2.36 ± 1.25
It is easy to decide myself how many cigarettes I want to smoke	2.57 ± 1.13	2.60 ± 1.14
It is easy to say no if someone offers me a cigarette	2.49 ± 1.09	2.55 ± 1.10
It is easy not to smoke when I am stressed	1.86 ± 0.86	1.94 ± 0.89
It is easy not to smoke when I drink alcohol	2.30 ± 1.14	2.35 ± 1.12
It is easy not to smoke when I drink coffee or tea	2.90 ± 1.25	3.01 ± 1.22
It is easy not to smoke when I go to a cafe or restaurant	2.89 ± 1.20	2.93 ± 1.17
It is easy not to smoke after a meal	2.33 ± 1.08	2.47 ± 1.11
**Habit**[6]		
Average score of scale [α 0.729]	3.76 ± 0.88 (5716)	3.59 ± 0.91 * (1124)
Smoking is something I do automatically	4.12 ± 0.84	4.04 ± 0.88
Smoking is something I do without thinking	3.81 ± 1.05	3.59 ± 1.10
Smoking is something I start doing without realizing I'm doing it	3.35 ± 1.17	3.14 ± 1.17
**Identity**		
Average score of scale [α 0.718]	2.96 ± 0.76 (5728)	3.06 ± 0.80 * (1127)
I feel connected to non-smokers	2.79 ± 0.86	2.89 ± 0.91
I feel at home in the company of non-smokers	3.12 ± 0.88	3.23 ± 0.88

*significant difference at $\alpha \leq 0.05$ for comparison between all baseline respondents and those who participated in the three-month follow-up [1]N: number, [2]M: mean, SD: standard deviation, [3]Primary education (basisschool), lower secondary education (BBL, KBL, VMBO), lower vocational education (LBO, [4] Middle or higher secondary education (HBS, HAVO, VWO), middle vocational education (MTS, MULO, MBO), [5] Higher vocational education (HBO), university, M: mean, SD: standard deviation, [6] Likert scale items were only reported by those who were current smokers at the three-month follow-up survey
